# Characterization of Sparkling Wines According to Polyphenolic Profiles Obtained by HPLC-UV/Vis and Principal Component Analysis

**DOI:** 10.3390/foods8010022

**Published:** 2019-01-10

**Authors:** Anaïs Izquierdo-Llopart, Javier Saurina

**Affiliations:** Department of Chemical Engineering and Analytical Chemistry, University of Barcelona, Martí i Franquès 1-11, 08028 Barcelona, Spain

**Keywords:** liquid chromatography, polyphenols, protected designation of origin, coupages, sparkling wine (cava), characterization, chemometrics

## Abstract

Cava is a sparkling wine obtained by a secondary fermentation in its own bottle. Grape skin contains several compounds, such as polyphenols, which act like natural protectors and provide flavor and color to the wines. In this paper, a previously optimized method based on reversed phase high performance liquid chromatography (HPLC) with ultraviolet/visible (UV/Vis) detection has been applied to determine polyphenols in cava wines. Compounds have been separated in a C_18_ core-shell column using 0.1% formic acid aqueous solution and methanol as the components of the mobile phase. Chromatograms have been recorded at 280, 310 and 370 nm to gain information on the composition of benzoic acids, hidroxycinnamic acids and flavonoids, respectively. HPLC-UV/vis data consisting of compositional profiles of relevant analytes has been exploited to characterize cava wines produced from different base wine blends using chemometrics. Other oenological variables, such as vintage, aging or malolatic fermentation, have been fixed over all the samples to avoid their influence on the description. Principal component analysis and other statistic methods have been used to extract of the underlying information, providing an excellent discrimination of samples according to grape varieties and coupages.

## 1. Introduction

Cava is a sparkling wine of high quality with Protected Designation of Origin (PDO) produced by the Champenoise method based on the second fermentation and aging period in its own bottle [1,2]. Cava is highly popular in our society: it is currently the most exported Spanish wine. For this reason, control strategies are needed to guarantee the standards of quality and the maintenance of organoleptic characteristics among winemaking batches. Part of this evaluation is carried out by sensory analysis with a group of expert panelists. Alternatively, analytical methods can be implemented to satisfy the increasing demand of controls, to reduce costs and to achieve reliable results [3]. The study of polyphenols in wines is a hot topic in the field of food analysis, because of the implications on organoleptic and descriptive issues [3,4]. For instance, the role of anthocyanins as principal pigments of wine or the influence of tannins on bitterness and astringency are well-known, while, apparently, they have a little direct contribution to odor [5].

Red wines from black skin grapes are much richer in polyphenols than rosé or white wines due to the simple fact that these compounds are extracted from the skin grape during the maceration process. Furthermore, cavas produced from the Chardonnay variety have a high content in polyphenolic substances; however, they differ in composition from those found in the varieties of black grapes [6]. Moreover, polyphenols are the substrate for some enzymatic changes of must and the main responsible for non-enzymatic auto-oxidation processes of the wines. Polyphenols have also shown a great interest as nutraceuticals because of the wide range of beneficial effects attributed, including antioxidant, antimicrobial and anti-inflammatory activities [7,8]. 

It has been pointed out elsewhere that naturally occurring components of food product may serve as efficient descriptors of some food features such as origin, varietal constituents, manufacturing practices, etc. [3,9]. Among the different food matrices, wines have been studied extensively on the basis of contents of chemical species, such as volatile compounds [10], inorganic elements [11,12], organic acids [13], amino acids and biogenic amines [14], polyphenols [15,16], etc. The corresponding compositional profiles and instrumental fingerprints have been used as the source of chemical information [17] to tackle characterization, classification and authentication issues, often with the assistance of chemometric methods. 

In the field of cava and wines, polyphenolic data can be correlated with factors (e.g., geographic origin, climate and terrain, grape varieties, time of harvest and winemaking procedures) with a high impact on quality and economic issues, such as the qualification with the PDO or the final price [6]. Some applications have been reported on the role of polyphenols and related compounds as descriptors of cava features. It should be remarked that, in some of the cases mentioned below, principal component analysis (PCA) and related chemometric methods (cluster analysis, discriminant analysis, etc.) were used to facilitate the extraction of relevant information. A pioneering publication evaluated the descriptive ability of main components of base white wines from Macabeu, Xarel·lo and Parellada grapes to infer on their discrimination according to varieties, vintages or wineries; in the study, authors pointed out the possibilities of species such as cinnamic acids as potential varietal markers [18]. In another case, Martinez-Lapuente et al. studied the effect of alternative grape varieties on the polyphenolic composition and sensorial features of sparkling wines [19]. Patters on color and taste attributes and overall quality depending on varieties were drawn. It was concluded that Alvaring and Verdejo wines resulted in highly attractive alternatives. In a recent paper, the effect of some oenological practices on the composition of phytochemicals of rosé sparkling wines was evaluated; the influence of some additives to preserve the levels of the most abundant polyphenols was assessed as well [20]. Stefenon and coworkers investigated the influence of aging on lees on the polyphenolic content and antioxidant activity of sparkling wines obtained under Champenoise and Charmat winemaking protocols [21]. The authors found out changes in the evolution of the contents of species, such as tirosol, gallic acid, resveratrol or piceid, which varied differently depending on the vinification method. Another study compared some parameters of Champenoise, Charmat and Asti methods on the aromatic composition of Moscato Giallo sparkling wines. The authors concluded that the total polyphenolic content and the in vitro antioxidant activity of samples did not show significant differences among the three vinifications [22]. An investigation on biological aging and storage of cava wines revealed differences in the composition of browning components related to phenolic and furfural species as a function of the process time; the concentration of hydroxylmethylfurfural could be proposed as an index of cava quality [23]. A similar research also demonstrated the influence of oenological and aging processes on color changes and phenolic composition of rosé sparkling wines; in this case, color features could be associated to the transformation of polymeric species into absorbing anthocyanins as a consequence of aging [24]. Finally, Bosch-Fusté et al. investigated the viability of the total index of phenols defined as the absorbance at 280 nm as a quality marker of cava wines subjected to an accelerated aging process. The study of two sets of samples showed an increase in the phenolic index after seven weeks of accelerated breeding [25].

In this paper, relevant polyphenols were determined in different cava samples by an HPLC-UV/vis method previously established [26]. Compounds were separated chromatographically on a C_18_ column under an elution program based on increasing the percentage of methanol. In order to focus on varietal and blending issues more specifically, samples under study were chosen homogeneously regarding some winemaking factors such as vintage, aging in cellars and application of malolactic fermentation. As a result, descriptors of wine blending could be investigated without the influence of other disturbances. PCA was applied to compositional data to carry out an exploratory study of the coupages made with different grape varieties. Despite the lack of selective descriptors, results showed that various polyphenols were more abundant in some coupages. As a result, we concluded that the proposed method resulted in a simple, fast and suitable approach towards the characterization and classification of cavas according to base wine varieties and blends using polyphenolic concentrations as the source of information.

## 2. Materials and Methods

### 2.1. Chemicals and Solutions

The mobile phase was prepared with formic acid (>96%, Sigma-Aldrich, St. Louis, MO, USA), methanol (UHPLC-Supergradient, Panreac, Barcelona, Spain) and water (Elix3, Millipore, Bedford, MA, USA). Polyphenols of analytical quality were purchased from Sigma-Aldrich (St. Louis, MO, USA): gallic, homogentisic, protocatechuic, caftaric, gentisic, vanillic, caffeic, syringic, ferulic, *p*- coumaric acids, (+)-catechin, (−)-epicatechin, ethyl gallate, resveratrol, rutin, myricetin and quercetin. These compounds were selected according to their abundance and relevance in white and rosé cavas. Stock standard solutions of 1000 mg L^−1^ of each polyphenol were prepared in MeOH. Working standard solutions consisting of a mixture of analytes at concentrations ranging from 20 to 0.05 mg L^−1^ were prepared in MeOH:water (1:1, *v:v*).

### 2.2. Samples

Cava samples under analysis were kindly provided by the winery Codorníu Raventós S.A. Cavas were produced from base wines of Penedès and Costers del Segre regions (both from Catalonia, Spain). Samples consisted of white and rosé cavas all of them of 2015 vintage and aged for a period of 18 months, elaborated from various coupages of base wines of Chardonnay, Macabeu, Xarel·lo, Parellada, Pinot Noir, black Garnacha and Trepat varieties as follows: Coupage C (58 samples) corresponded to the three classical varieties: Macabeu, Xarel·lo and Parellada; Coupage E (six samples) was based on the classical combination with a small percentage of Chardonnay; Coupage A (15 samples) consisted of Chardonnay (70%) plus Macabeu, Xarel·lo and Parellada blending (30%); Coupage G (three samples) was Chardonnay 100%; Coupage S (two samples) was Chardonnay (50%) plus Macabeu and Xarel·lo (50%); Coupage T (four samples) corresponded to a rosé cava from Chardonnay (30%) and Pinot Noir (70%); Coupage V (nine samples) corresponded to a rosé cava from Pinot Noir, black Garnacha and Trepat. For each coupage, samples were independently produced (i.e., from different wine batches) and bottled. From the point of view of sugar content, the set comprised 11 brut nature (<3 g L^−1^ sugar), 61 brut (3–12 g L^−1^), 13 dry (17–32 g L^−1^) and 12 semi-dry (32–50 g L^−1^).

Samples were degasified and filtered through a nylon membrane (0.45 µm pore size) prior HPLC-UV/vis analysis. A quality control solution consisting of mixture of 50 µL of each cava sample was prepared to evaluate the reproducibility of the chromatographic method and the significance of the descriptive PCA models. Cava samples were analyzed randomly and the quality control was injected every 10 samples.

### 2.3. Chromatographic Method

The chromatographic system consisted of an Agilent Series 1100 HPLC Chromatograph (Agilent Technologies, Palo Alto, CA, USA) equipped with a quaternary pump (G1311A), a degasser (G1322A), an automatic injection system (G1392A) and a diode array detector (G1315B). An Agilent ChemStation for LC 3D (Rev. A. 10.02) software was used for instrument control and data processing.

The chromatographic method was optimized and validated elsewhere [26]. Briefly, the separation was carried out in a Kinetex C_18_ column (Phenomenex, Torrance, CA, 100 mm × 4.6 mm internal diameter with 2.6 μm particle size). 0.1% of formic acid in water (*v/v*) (solvent A) and methanol (solvent B) were used to create the following elution gradient and the flow rate was 1 mL min^−1^: from 0 to 20 min, B(%) 15–60 lineal increase; from 20 to 22 min, B(%) 60–90 lineal increase; from 22 to 27 min, B(%) 90 isocratic range, cleaning step; from 27 to 27.5 min, B(%) 90–15; from 27.5 to 30, B(%) 15 isocratic range, conditioning step. The injection volume was 10 µL. Chromatograms were acquired at 280, 310 and 370 nm.

### 2.4. Data Analysis

Preliminary statistics on polyphenolic contents in cava samples were gained from box and radial plots obtained with Excel. Exploratory studies by Principal Component Analysis (PCA) were carried out using the PLS-Toolbox working with MATLAB. The data matrix to be treated consisted of concentration values of quantified polyphenols in the cava samples. Data was autoscaled to achieve a similar weight to all the polyphenols regardless differences in amplitude and magnitude.

Sample patterns related to the different grape varieties and blends and other oenological practices were deduced form the interpretation of the scatter plot of scores of the first principal components (PCs). The distribution of variables on the space of the PC, shown in the plot of loadings, provided information on polyphenol correlations. Compounds up- or down expressed, which could be considered as tentative markers, were assessed from the simultaneous interpretation of scores and loadings graphs.

## 3. Results and Discussion

**Figure 1:**
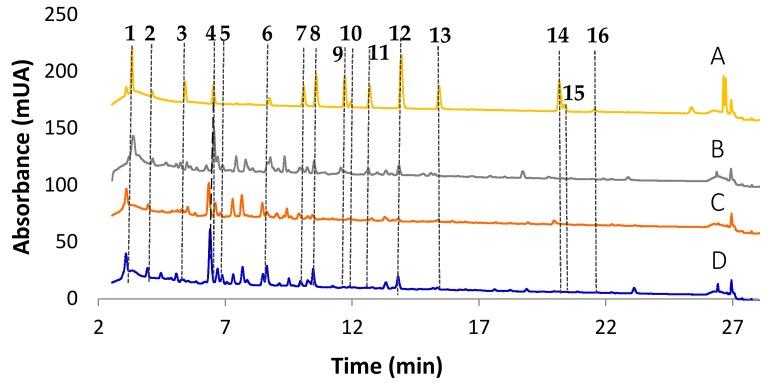
Chromatograms recorded at 280 nm using the proposed HPLC-UV/vis method. (**A**) Standard solution at 5 mg L^−1^; (**B**) Rosé wine composed of Pinot Noir, Trepat and black Garnacha varieties (**C**) Classical grape varieties coupage composed of Macabeu, Xarel·lo and Parellada and (**D**) White cava composed of Chardonnay variety. Peaks assignment: (1) gallic acid, (2) homogentisic acid, (3) protocatechuic acid, (4) caftaric acid, (5) gentisic acid, (6) catechin, (7) vanillic acid, (8) caffeic acid, (9) syringic acid, (10) ethyl gallate, (11) epicatechin, (12) *p*-coumaric acid, (13) ferulic acid, (14) resveratrol, (15) rutin and (16) myricetin.

As indicated above, an HPLC-UV/vis method previously developed for the determination of relevant polyphenols in white wines [26] was here extended to cava (sparkling) wines. Samples under study were similar with respect to various oenological features such as aging, vintage, malolactic fermentation or Champenoise vinification, thus, the variability was associated to the varietal origin of grapes and the combination of base wines. The three main cava classes corresponded to the so-called classical blend (Macabeu, Xarel.lo and Parellada), the rosé blend (Pinot Noir, black Garnacha and Trepat) and the Chardonnay monovarietal cava. The other cava types evaluated consisted of combinations of classical, rosé and Chardonnay wines in different percentages. Figure 1 shows the chromatograms of representative rosé, classical and Chardonnay samples recorded at 280 nm. The chromatogram of a standard mixture of polyphenol at 5 mg L^−1^ was also included for identification purposes. It can be seen that most of the selected compounds were detected although some differences in the concentration levels were encountered among coupages. Analytes were quantified by linear regression models stablish using 10 standard mixtures with concentrations ranging from 0.05 to 20 mg L^−1^. Differences in compositional profiles were the basis of further sample discrimination as a function of base wine varieties and coupages.

The analysis of the 97 samples under study revealed that, in general, hydroxycinnamic acids and their esters with tartaric acid were predominant species in this kind of winemaking process; benzoic acids were also abundant, while flavonoids were scarcer. As detailed in Table 1, wine samples were rich in gentisic and caftaric acids, with mean concentrations of 32.2 and 11.2 mg L^−1^, respectively. Gallic, homogentisic and caffeic acids, and catechin occurred at moderate levels, ranging from 1 to 5.5 mg L^−1^. Other minor components such as epicatechin, protocatechuic acid, p-coumaric acid vanillic acid and syringic acid were found at mean concentrations below 1 mg L^−1^. Resveratrol and flavonoids, such as rutin and myricetin, were scarce (not detected in all the samples). The descriptive ability of each polyphenol (i.e., the information contained in each variable) was accounted from the relative standard deviation of the concentration in the series of cavas. The variability ranges given in Table 1 indicated that concentrations of gallic and gentisic acids were quite homogeneous in all the samples regardless of grape varieties or coupages, with overall relative standard deviation (RSD) values below 10%. This finding suggested that the apparent discriminant capacity of these polyphenols to classify cava wines was certainly limited. In contrast, contents of compounds such as epicatechin, protocatechuic acid, vanillic acid, ferulic acid and syringic acid were more heterogeneous, with RSD (%) values higher than 30%. As a result, we guessed that they could result in biomarker candidates to distinguish the different cava classes.

**Figure 2:**
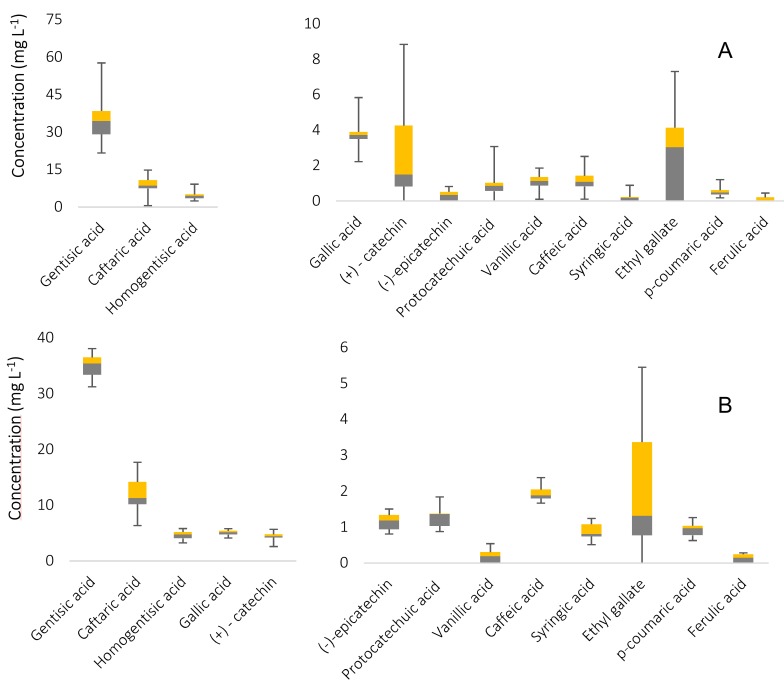
Boxplots with whiskers with the polyphenolic composition of the sets of (**A**) white and (**B**) rosé cava samples under study. Error bars indicated the variability in the concentration values.

Boxplots and radial diagrams were used to explore the performance of polyphenols to distinguish cava as a function of coupages. Figure 2 depicts the overall analyte content and mean concentration values of analytes in the white and rosé cava classes. 

**Figure 3:**
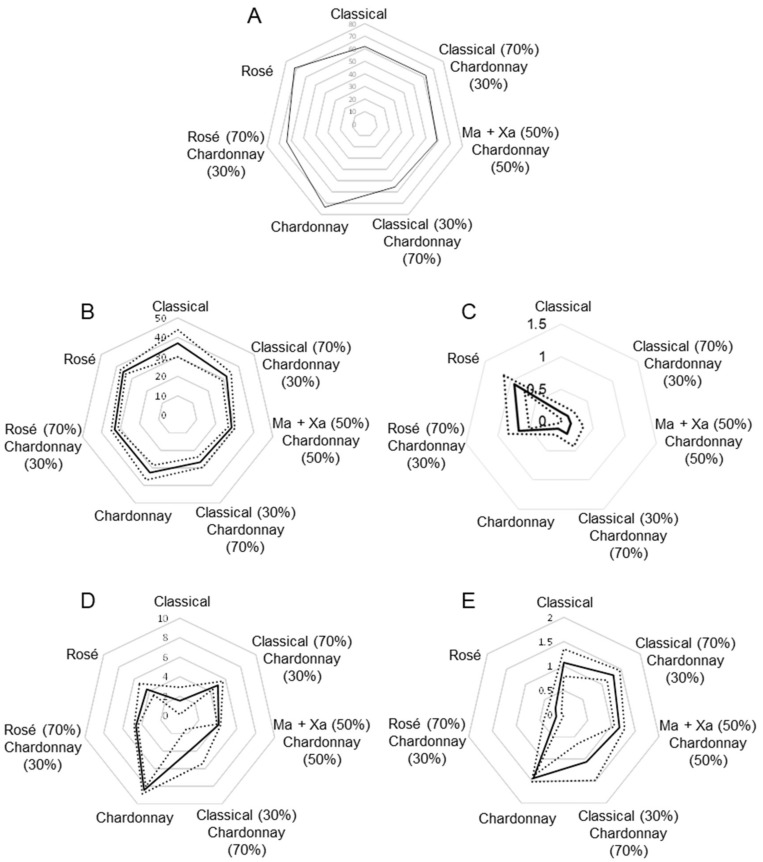
Radial plots of polyphenolic concentrations in the different coupages. (**A**) Overall content of analytes; (**B**) gentisic acid; (**C**) syringic acid; (**D**) catechin; (**E**) vanillic acid. Solid line indicates the mean value; dotted lines indicated the ± standard deviation values. Variety assignation: Ma, Macabeu; Xa, Xarel·lo.

In general, rosé (Pinot Noir, Trepat and black Garnacha combinations) and Chardonnay cavas showed the richest overall polyphenol contents, while the classical bends of Macabeu, Xarel·lo and Parellada cavas contained lower polyphenol levels (Figure 3A). In accordance with our previous results, gentisic acid was homogenously distributed among classes (Figure 3B); concentrations of the vast majority of samples ranged between 25 and 35 mg L^−1^. This compound was not up- or down-expressed in any particular variety, thus it resulted in a poor descriptor. Additionally, gallic acid was widespread, although its concentration was slightly increased in rosé samples with Pinot Noir and black Garnacha varieties. Syringic acid was important in rosé cavas while it was quite residual in classical and Chardonnay-based cavas (Figure 3C); a similar pattern was observed for protocatechuic acid and epicatechin. Catechin (Figure 3D) and homogentisic acid were more abundant in Chardonnay and occurred at low concentrations in the coupages rich in classical varieties; as a result, catechin contents were highly dependent on the percentages of this variety in the samples. Another interesting pattern corresponded to vanillic acid which was present at low concentrations in rosé cavas (ca. 0.2 mg L^−1^) and reached 5- to 10-fold higher concentrations in the other classes (Figure 3E). All the hydroxycinnamic acids (caffeic, ferulic, coumaric, etc.) behaved in a similar way, being more abundant in Chardonnay and rosé samples. Other compounds were less relevant for descriptive purposes as they displayed a less systematic behavior.

### Principal Component Analysis

Compositional profiles were used as the data to be treated by PCA for a more comprehensive study focused on identifying potential markers of the different classes of wine varieties and blends. The dataset consisted of concentration values of each polyphenol in the cava samples and the quality controls (QCs). As remarked in the experimental section, QCs were included to assess the soundness of the model. A first exploration carried out on the whole data matrix indicated that samples were reasonably distributed according to the coupages. Some minor components, such as resveratrol, rutin and myricetin, with concentrations close to the detection limits of the method, displayed high variability (RSD values in the QC replicates higher than 20%); thus, they were excluded from the dataset to obtain a more robust and accurate description of sample behavior. The rest of the variables, which exhibited RSD values lower that 6%, were taken for analysis.

**Figure 4:**
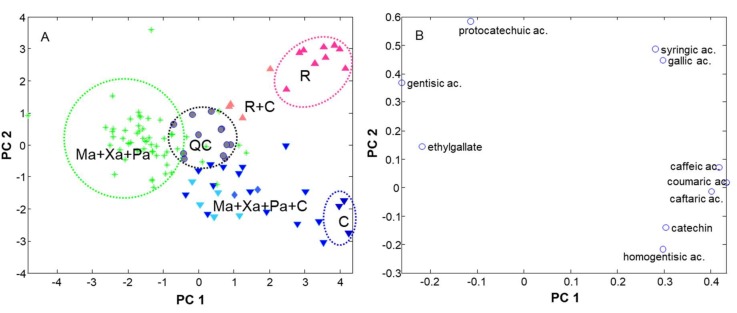
Principal component analysis of the dataset consisting of polyphenol concentrations of each cava sample. (**A**) Plot of scores of PC1 versus PC2; (**B**) plot of loadings of PC1 versus PC2. Acronyms: C Chardonnay; Ma Macabeu; Pa Parellada; Xa Xarel·lo; QC Quality control; R rosé. Symbols: Star = classical coupage (Ma + Xa + Pa); Triangle (vertex up) = rosé cava; Triangle (vertex down) = Chardonnay cava; circle = QC.

The PCA model working with the refined set indicated that circa 75% of the information was captured with three principal components (PCs). In particular, PC1 retained 41.3% of variance, PC2 23.3% and PC3 9.9%. Scatter plots of scores of the relevant PCs revealed interesting patterns on sample distribution. In the case of PC1 versus PC2 (Figure 4A), QCs were located in a compact group in the central area of the graph, thus proving that the overall model was reliable and robust.

Interestingly, PC1 modeled the overall polyphenolic content with the richest cavas on the right and the poorest on the left. PC2 explained differences among rosé, classical and Chardonnay (top, center and bottom, respectively). More specifically, three main trends in the sample distribution were encountered, namely: (i) cavas in the left side were mainly elaborated with classical grape varieties of Macabeu, Xarel·lo and Parellada; (ii) samples in the bottom-right corner corresponded to monovarietal Chardonnay; (iii) rosé cavas prepared with black grapes appeared in the top-right corner. Intermediate situations of coupages elaborated with variable percentages of Chardonnay (e.g., classical varieties combined with 30, 50 or 70% of Chardonnay or rosé varieties combined with 30% Chardonnay) were distributed accordingly (see Figure 4A). Finally, although not shown here, PC3 mainly modeled the variations of Chardonnay proportions in the coupages, with larger scores for those samples containing higher percentages.

The scatter plot of loadings of PC1 versus PC2 (Figure 4B) described the behavior of the polyphenolic variables. It was first deduced that the levels of some compounds were reasonably correlated, e.g., syringic and gallic acids, and caffeic and coumaric acids, with correlation coefficients higher than 0.6. Other species were more negatively correlated (e.g., ethyl gallate and caftaric acid). The simultaneous study of scores and loadings indicated that classical cava coupages contained higher amounts of gentisic acid and ethyl gallate as they occupied the left side of the graphs. Syringic and gallic acids were representative of rosé cavas elaborated with red grapes varieties of Pinot Noir, Trepat and red Garnacha. Finally, catechin, homogentisic, caftaric, caffeic and coumaric acids were more abundant in Chardonnay cavas and coupages created with high percentages of this variety.

Apart from the relationship between phenolic matter and grape varieties, the sugar content was another variable of the set of samples under study. The liquor of expedition after bottle disgorging provided different concentrations of sugar, ranging from 50 g L^−1^ for semi-dry to less than 3 g L^−1^ for brut nature. In the current case, PCA plots showed that the sugar content was uncorrelated with polyphenols and had no influence on the sample distribution. Regarding geographical factors and agricultural practices that may affect substantially the phenolic levels, their effect on the set of samples under study was buffered, since enologists combined appropriate proportions of base wines from different regions to obtain products with more constant physicochemical and sensorial attributes, according to commercial parameters of quality and the costumer’s preferences. Finally, as indicated above, other variables such as vintage and aging, which may notably influence on the polyphenolic composition, were controlled for; thus, patterns observed mainly depended on the combinations of base wines.

## 4. Conclusions

Sparkling wines belonging to the protected designation of origin Cava were analyzed with a chromatographic method for the determination of polyphenols. Wines from different coupages were considered including monovarietal Chardonnay samples, rosé samples composed of Pinot Noir, Trepat and black Garnacha, the classical blends of Macabeu, Xarel·lo and Parellada and other mixtures containing variable amounts of Chardonnay. Other oenological variables, such as vintage, aging and malolactic fermentation, were fixed for all the samples so that they did not affect the description. In general, overall polyphenolic contents were higher in Chardonnay wines, followed by rosé, while the classical coupages were circa 20% poorer in these components. Patterns deduced from boxplots and radial diagrams were confirmed from a comprehensive data exploration by principal component analysis. Although no molecule was found to be a specific descriptor of cava classes, syringic acid was more abundant in rosé wines, the hydroxycinnamic acids were up-expressed in Chardonnay samples and vanillic acid was almost residual in red grape products. Summarizing, polyphenolic profiling combined with a chemometric data analysis resulted in an excellent approach to deal with the characterization of cava wines according to the coupages. This strategy could be extended to further classification and authentication purposes.

## Figures and Tables

**Table 1:** Average concentration values of polyphenols in the set of samples under study.

Compounds	Average Concentration (mg L^−1^)	SD	RSD (%)
Gallic acid	4.1	0.3	7.0
Homogentisic acid	5.2	0.8	15.5
Protocatechuic acid	0.7	0.2	31.4
Caftaric acid	11.2	1.2	13.6
Gentisic acid	32.2	3.2	9.7
Catechin	4.5	0.8	28.1
Caffeic acid	1.5	0.2	14.5
*p*-Coumaric acid	0.74	0.13	19.3
Vanillic acid	0.92	0.21	46.5
Syringic acid	0.34	0.16	76.9
Epicatechin	0.60	0.21	66.8
Ferulic acid	0.12	0.14	120
Resveratrol	0.04	0.07	170
Rutin	0.007	0.035	165
Myricetin	0.029	0.089	92.0

Standard deviation (SD) and relative standard deviation (RSD) indicated the variability of concentrations as a measure of discriminating capacity among samples.
